# Influence of glucose as a natural reductant on silver nanoparticles synthesis for decontamination of *p*-nitrophenol and methylene blue from wastewater

**DOI:** 10.1038/s41598-025-11974-z

**Published:** 2025-07-25

**Authors:** Ayman H. Mansee, Doaa M. Abdelgawad, Amal M. Ebrahim

**Affiliations:** 1 Department of Pesticide Chemistry & Technology, Faculty of Agriculture, Alexandria University, Alexandria, Egypt; 2 Department of Soil & Water Science, Faculty of Agriculture, Alexandria University, Alexandria, Egypt

**Keywords:** Wastewater treatment, Catalytic reduction, Green silver nanoparticles, Glucose, Chemisorption, *p*-Nitrophenol, Methylene blue, Pollution remediation, Environmental sciences

## Abstract

Silver nanoparticles (Ag°/glucose) were synthesized based on glucose as a natural reducing agent, aiming to develop an eco-friendly catalytic system. The characteristics of the produced Ag°/glucose were confirmed using standard nanomaterial characterization techniques. The optimum conditions for eliminating *p-*nitrophenol (PNP) and methylene blue (MB) from artificial polluted water using Ag°/glucose were systematically explored. Various kinetic and isotherm models were applied to elucidate the sorption mechanism and behavior. The synthesized Ag°/glucose exhibited a surface plasmon resonance (SPR) peak at 430 nm, with an average particle size ranging from 21 to 31 nm, and a zeta potential recorded as − 16 mV. The final concentration of Ag°/glucose was determined to be 1.2 × 10^− 6^ mol/L. During the first 15 min of incubation, a dose of 20 µL/mL Ag°/glucose achieved 53% and 74% removal of the targeted PNP and MB, respectively. Increasing the dose to 30 µL/mL showed a complete removal of both pollutants. Kinetic analysis revealed that the pseudo-second-order model was the best fitting model for both PNP and MB adsorption processes. Isothermal data showed a superior appropriateness of the present results to the Langmuir and Freundlich model for describing sorption behavior, with maximum adsorption capacities (*q*_*max*_) of 2.5 E + 3and 1.0 E + 3 mg/g for PNP and MB, respectively.

## Introduction

With the increasing progress of industrialization and rapid urban development, a growing volume of hazardous wastewater is being discharged into the environment, particularly into water bodies, with minimal or no treatment. Clean water is essential for sustaining life; however, maintaining its quality for various applications has become increasingly challenging due to the significant amounts of waste generated from industrial, municipal, and residential sources^1^. Recently, the discharge of organic dyes and nitro compounds into aquatic environments has emerged as a significant environmental concern, as these toxic chemicals pose a serious threat to all forms of life on the planet. Furthermore, dyes like MB are examples of non-biodegradable organic pollutants that have an adverse effect on water quality by producing an unpleasant odor, blocking light penetration, raising the demand for chemical oxygen, lowering the concentration of dissolved oxygen, and ultimately killing aquatic life^2^. Therefore, efficient and pointed manners for removing organic dyes from water systems is a critical worldwide concern for treating wastewater^3^. Also, one of the worst types of organic contaminants originating from industrial and agricultural activities is the *p*-nitrophenol (PNP), which is listed by the US Environmental Protection Agency^4,5^ as one of 129 compounds that may cause cancer.

Reverse osmosis, photochemical, biological, coagulation-flocculation, chemical oxidation, adsorption, membrane separation, electrochemical, aerobic, and anaerobic microbial degradation are some of the different techniques for removing organic pollutants. Adsorption is the most successful physicochemical technique for eliminating organic pollutants from wastewater, hence it involves using of a variety of sorbed materials^1,6,7^.

In the environmental pollution domain, nanotechnology has several applications, such as cleanup, monitoring, detection, and prevention^8^. Nanomaterials can be used to get rid of these pollutants because of their unique properties. This technology is inexpensive, safe, and ecologically benign. Numerous research on the potential of nanomaterials for water body remediation has been conducted recently. Many promising nanoparticles, including nickel oxide^9^ carbon nanotubes^10^ silver nanoparticles^6^ and zero-valent iron^11^ have been employed in wastewater treatment.

Compared to those of typical materials, nanostructures, especially silver nanoparticles, have a larger surface area, which enhances their potential for environmental remediation and lowers the overall cost of eliminating pollutants^12,13^. Therefore, silver nanoparticles are excellent catalysts for a variety of catalytic reduction processes^14,15^.

Silver nanoparticles were synthesized according to different reduction approaches, depending on chemicals, physical, or green reducing agents like plant extract, ascorbic acid, sodium borohydride, hydrazine, sodium citrate, glucose, and polyvinyl alcohol. The presence of natural antioxidants, such as alkaloids, phenols, citric acid, polyphenols, terpenes, ascorbic acid, flavonoids, proteins, amino acids, carbohydrates, saponins, flavonoids, chromones, steroids, saturated-unsaturated fatty acids, terpenoids, and other components, is crucial for the synthesis process, and essential for improving the physical and chemical properties of the Ag nanoparticles^16 –19^. This is one of the most important reasons for the efficiency variances of different formulas in their catalytic activities. For example, green silver nanoparticles observed successful removing for methylene blue and *p*-nitrophenol^4,20^ methyl orange, methyl red, and congo red^21^ and hexavalent chromium^6^ from contaminated water.

In the present study, silver nanoparticles were greenly synthetized depending on D-glucose for reducing silver ions to metallic silver, and through this process, it oxidizes itself to gluconic^22,23^. Here, the present study aimed to: (1) greenly synthesis Ag° using glucose as a natural reductant, (2) examine the Ag°/glucose catalytic activity and figured out the optimum operating conditions for removing PNP and MB, and (3) conducting kinetic and isothermal studies to explore the removal mechanism and behavior.

## Materials and methods

### Ag°/glucose green synthesis and characterization

Ag°/glucose was synthesized using the procedure previously outlined by Darroudi et al.^22^ as follows: (1) 1% gelatin was dissolved in 10 mL of AgNO_3_ (1 M) solution. (2) 10 mL of NaOH (1 M) was added to the AgNO_3_/ gelatin solution, (3) 10 mL of a glucose solution (2 M) was added to the mixture after it had heated up to 60 °C, and the reaction was then allowed to proceed for 15 min.

Several routine tests were conducted to confirm the success of the green synthesis process^21^:

1. Primarily, the reduction process was seen visually by observing the color shift from yellow to brownish yellow to deep brown.
2. An Alpha 1502 UV-visible spectrophotometer (Laxco, Inc., Bothell, WA 98021, USA) was used to scan the obtained Ag°/glucose at 50 nm intervals between 250 and 750 nm in order to verify the reduction process.
3. Using scanning electron microscopy (MODEL JSM-IT200), the Ag°/glucose surface shape and particle size were investigated and characterized.
4. FTIR spectroscopy was used to investigate the chemical components that led to the reduction of silver ions and the capping agent of silver nanoparticles (PerkinElmer Spectrum IR Version 10.6.0).
5. The Ag°/glucose zeta potential was examined using the Malvern Zeta Sizer.
6. The Ag°/glucose final concentration was theoretically calculated^24^.

### Batch sorption experiments

The best operating parameters for removing PNP (99% purity) and MB (70% purity) in a single liquid state from artificially polluted water by employing Ag°/glucose as a sorbent material were investigated by a set of sorption studies^3,20,25^. In these tests, the concentration of pollutants (2, 5, and 10 µg/ml) was examined, along with the contact time (15–60 min) and Ag°/glucose dose (10, 20, and 30 µL/mL). Every test was conducted three times, and the mean values were used as the results. For the PNP samples, 300 µL/mL NaBH_4_ (0.5 mM) was added. The PNP and MB removal percentages (Eq. 1) and quantity of sorbed pollutant (Eq. 2) were determined using a UV–Visible spectrophotometer at 400 and 665 nm, respectively, and calculated according to equations illustrated in Table 1.

**Table 1:** Summary of the mathematical equations used in this study for data analysis.

Models	Equation	Parameters
Removal Percentage, Eq. 1	$\:\text{R}\:\left(\text{\%}\right)=\frac{{C}_{i}-{C}_{f}}{{C}_{i}}\times\:100$	C_i =_ initial concentration (µg/mL)
		C_f =_ final concentration (µg/mL)
adsorbed amount *q*_*e*_, Eq. 2	$\:{q}_{e}=\frac{\text{V}\:({C}_{i}-{C}_{f})}{m}\:\:\:\:\:\:$	**V =** the volume of reaction solution (L)
		m = is the weight of Ag°/glucose (g).
Pseudo-first order, Eq. 3	$\:\text{l}\text{n}\left({\text{q}}_{\text{e}}-{\text{q}}_{\text{t}}\right)=\:\:\:\text{l}\text{n}{\text{q}}_{\text{e}}\:-{\:\text{k}}_{1}\text{t}$	q_e_ = the adsorbed pollutant per gram of Ag°/glucose at equilibrium (mg/g).
		q_t_ = the adsorbed pollutant per gram of Ag°/glucose at time t (mg/g).
		k_1_ = the rate constant for the pseudo-first-order model (min^− 1^).
Pseudo-second order, Eq. 4	$\:{\text{t}/\text{q}}_{\text{t}}=\:\:\frac{1}{{\text{k}}_{2}{\text{q}}_{\text{e}}^{2}}+\frac{1}{{\text{q}}_{\text{e}}\text{t}}$	k_2_ = the rate constant of the pseudo-second-order model (g/mg. min).
Elovich, Eq. 5	$\:{\text{q}}_{\text{t}}=\:\:{\upbeta\:}\:\text{l}\text{n}\left({\upalpha\:}{\upbeta\:}\right)+{\upbeta\:}\:\text{l}\text{n}\text{t}$	*α* and β *=* constants of Elovich model.
Intra-particle diffusion, Eq. 6	$\:{\text{q}}_{\text{t}}={\:\text{k}}_{\text{d}\text{i}\text{f}\text{f}}\:{\text{t}}^{0.5}+\text{C}$	k_diff_ = Intra-particle diffusion rate constant, (mg/mg. min)
		C = the thickness of boundary layer
Langmuir, Eq. 7	$\:\frac{{C}_{e}}{{q}_{e}}=\frac{1}{{q}_{max}}{C}_{e}+\frac{1}{{K}_{L}\:{q}_{max}}$	*q*_*e =*_ the amount of adsorbed per gram of sorbed at equilibrium (mg/g)
		*Ce =* equilibrium concentration of pollutant in the solution (mg/L)
		*q*_*max*_ *=* the maximum adsorption capacity of the Ag°/glucose(mg/g)
	$\:{R}_{L}=\frac{1}{1+{K}_{L\:}{C}_{0}}\:\:\:\:\:$	*K*_L_ = the Langmuir constant (L/mg)
Freundlich, Eq. 8	$\:\text{l}n\:{q}_{e}=\frac{1}{n}ln{C}_{e}+ln{K}_{f}\:\:$	*K*_*f =*_ the Freundlich constant (mg/g)
		*n* = Freundlich exponent
Temkin, Eq. 9	$\:{q}_{e}=B\:ln{C}_{e}+B\:ln{K}_{T}\:\:\:\:\:\:\:\:\:$	*B* and *K*_*T*_ = the Temkins’ model parameters (g/L)

### Kinetics model-based data analysis

Four kinetic models (Table 1) were used to properly explain the PNP and MB adsorption mechanism: the pseudo-first order (Eq. 3), pseudo-second order (Eq. 4), Elovich (Eq. 5), and intra-particle diffusion (Eq. 6) models^26,27^. Since the adsorption features of both pseudo-first order and pseudo-second order methods partially explain the adsorption mechanism, they do not offer sufficient systematic values. Considering the complexity of the kinetic process, it is necessary to evaluate several models, particularly the Elovich model and intra-particle diffusion, in order to get an accurate and thorough assessment of the entire kinetic investigation^28–31^.

### Isothermal model-based data analysis

In this section the PNP and MB sorption equilibrium data were applying to the linear form of three isothermal models (Table 1): Langmuir (Eq. 7), Freundlich (Eq. 8), and Temkin (Eq. 9) adsorption isotherm models allowed for the provision of important insights into the surface properties and affinity of the sorbent^6^.

## Results and discussion

### Ag°/glucose synthesis and characterization

Ag°/glucose nanoparticles were synthesized using a natural polymeric matrix, silver nitrate (silver precursor), gelatin (stabilizer), glucose (redacting agent), and sodium hydroxide (accelerator). Once silver ions were distributed throughout the gelatin matrix, the process of reduction was carried out as follows: gelatin reacted with Ag^+^ to create a stable silver-gelatin complex [Ag(gel)]^+^, which then reacted with OH^−^ to form silver metal because the reduction of silver ions was caused by the oxidation of glucose to gluconic acid^22^. This mechanistic insight was explained other studies, colloidal silver nanoparticles was synthesized using ‘green’ reducing agents either from different types of honey, or β-d-glucose. They explain the mechanism of the synthesis process as follows reduction of Ag + is assisted by the addition of NaOH. The alkaline environment facilitates the opening of the glucose ring by the abstraction of the α-proton of the ring oxygen, and following glucose is oxidized to gluconic acid^32^. Also, glucose can be oxidized to gluconic acid and reduce silver cations to AgNPs in an alkaline medium, and this mechanism is accelerated by heating^33^. As mentioned by Darroudi et al.^22^ the possible chemical equation for preparing the Ag-NPs is:

$$\:{Ag}_{aq}^{+}+{gel}_{aq}\:\:\to\:\:[{Ag\left(gel\right)]}_{aq}^{+}\:\:\:\:\:\:\:\:\:$$

$$\:2[{Ag\left(gel\right)]}_{aq}^{+}+\:{2OH}^{-}+\:{C}_{5}{H}_{11}{O}_{5}-CHO\:\to\:\:2Ag+\:2gel+{H}_{2}O+\:{C}_{5}{H}_{11}{O}_{5}-COOH\:\:\:$$

**Fig. 1:**
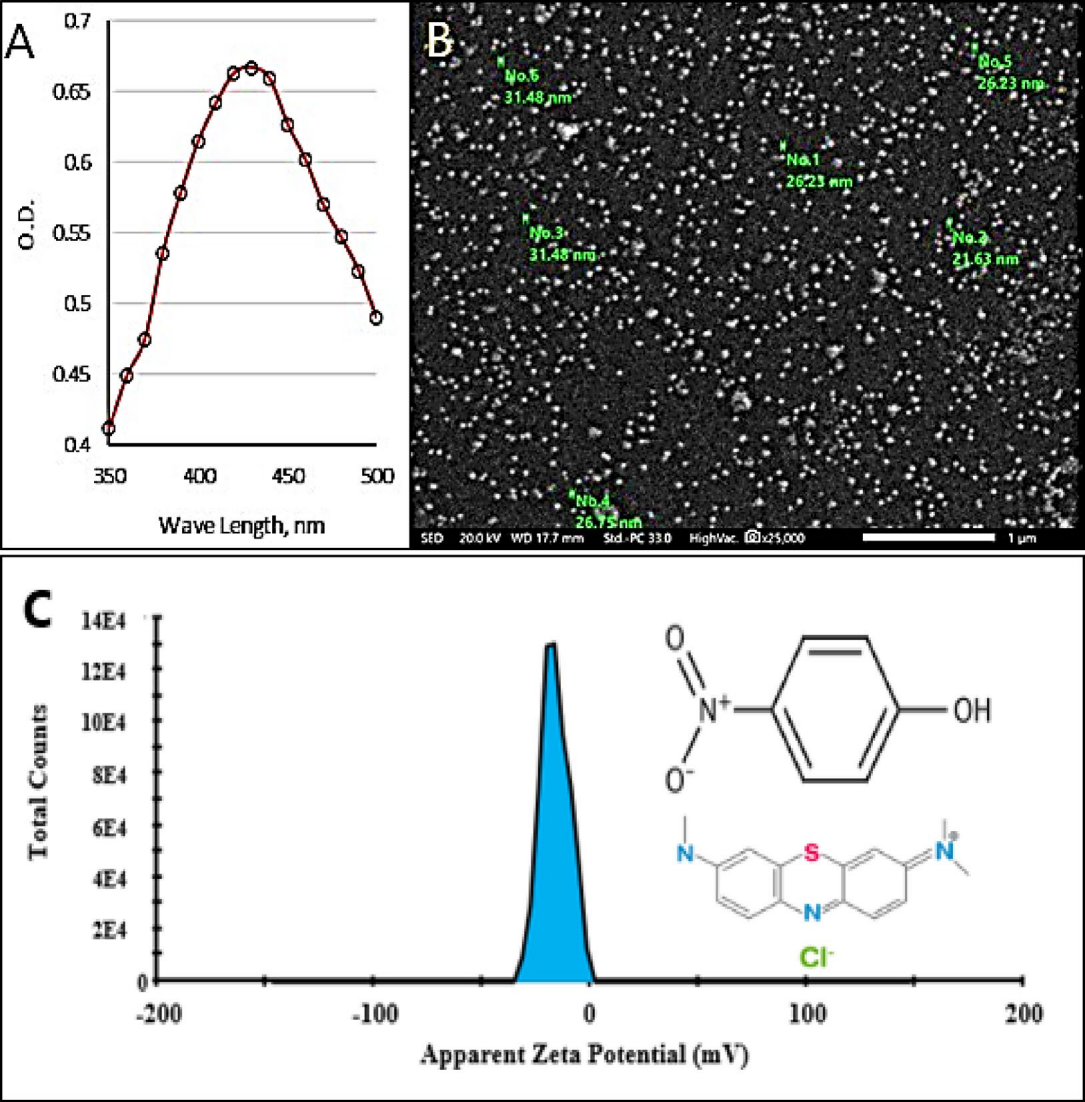

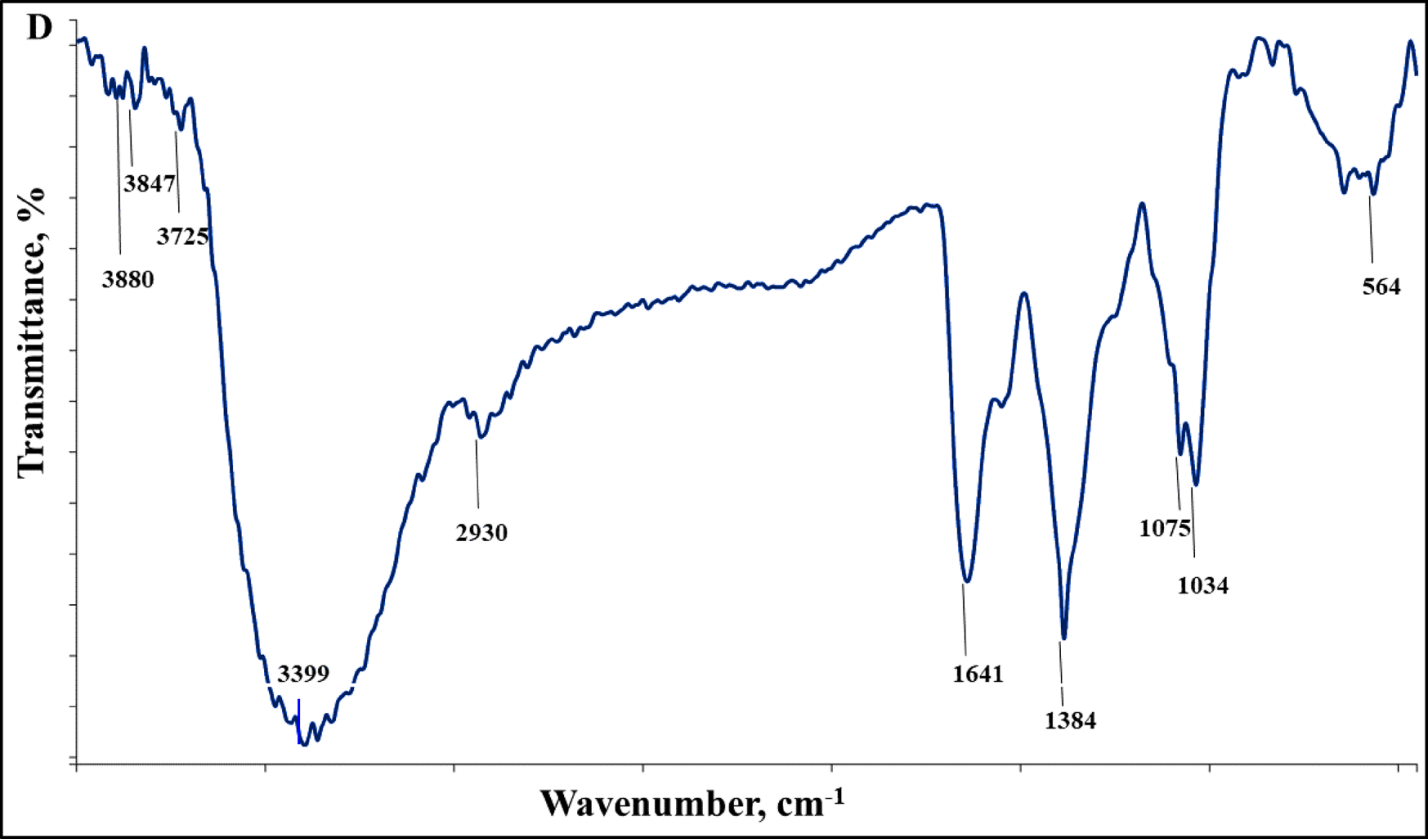
Ag°/glucose UV‒Visible spectra (**A**), SEM image (**B**), Zeta potential (**C**), and FTIR (**D**).

Surface plasmon resonance data from the spectroscopic scan (Fig. 1a) and visual inspection were utilized to verify the synthesis and stabilization of the green-synthesized Ag°/glucose. Previous studies observed changes in the color of Ag° colloidal solutions from yellow to dark brown due to the excitement of Ag° surface plasmon resonance. Such color transformation indicates a uniform dispersion of spherical Ag° particles^34,35^ where the absorption peaks ought to be situated between 400 and 450 nm^21^. A sharp plasmon with a λ_max_ at 430 nm appeared for the synthetized Ag°/glucose nanoparticles, is reported. Ag°/glucose’s surface morphology was examined using a scanning electron microscope (SEM) at a magnification of 35,000X and a 500 nm scale (Fig. 1b). The results demonstrated that the particle sizes of Ag°/glucose ranged from 21 to 31 nm, and the morphology form was almost spherical shape. The surface potential known as zeta potential is linked to the surface electrical charge and influences various aspects of material particles in suspension, such as surface contact, precipitation, and particle complexation^36^. The zeta potential of Ag°/glucose was recorded using Malvern Zeta Sizer instrument to measure the effective electric charge on the nanoparticle surface. Whereas, the zeta potential value is a crucial particles characteristic as it can influence both particles stability and properties. Theoretically, more pronounced zeta potential values, being positive or negative, tend to stabilize particle suspension. The electrostatic repulsion between particles with the same electric charge prevents the aggregation of the sphere^37–39^.

The recent data (Fig. 1c) showed that the Ag°/glucose nanoparticles have a negative surface charge of − 16mV. Remarkably, Ag°/glucose’s negative charge and ability to stabilize dispersed particles while preventing the formation of aggregates or precipitations seem to make it advantageous for uptake of cationic contaminants such as PNP and MB^40^. The FTIR spectrum of D-glucose shows the existence of a strong and broad absorption peak at 3391 cm^− 1^indicating the presence of v(OH) group stretching vibration. A small peak at 2920 cm^− 1^ was attributed to the absorption peak of v(CH2) group, and the bands at 1475 cm^− 1^ and 1328 cm^− 1^ were assigned to the bending vibration of v(CH). The v(C–O) and v(C–C) stretching bands were observed at 1132 and 1007 cm^− 1^, respectively^41^. While after the green synthesis of Ag°/glucose the FTIR spectrum shows different peaks at 3879.83, 3846.88, 3725.18, 3398.68, 2929.67, 1640.66, 1383.90, 1075.44, 1034.11, and 563.68 cm^− 1^ (Fig. 1d).

### *p-*nitrophenol and methylene blue removal

The two studied organic aromatic compounds PNP and MB have significant harmful effects on the aquatic ecosystem. As these compounds are essential to produce numerous medications, insecticides, phytochemicals, synthetic colors, and pharmaceutical raw materials, they are widely utilized and dispersed in laboratories and industrial processes. Because such compounds are difficult to decompose, even extremely low concentrations of them pose substantial hazards to aquatic habitats^42^. In this study, the effects of different major parameters (Ag°/glucose dose, contact time, and pollutant concentrations) on either PNP or MB removal efficiency from synthetic contaminated water were extensively investigated.

### Effect of Ag°/glucose dose

**Fig. 2:**
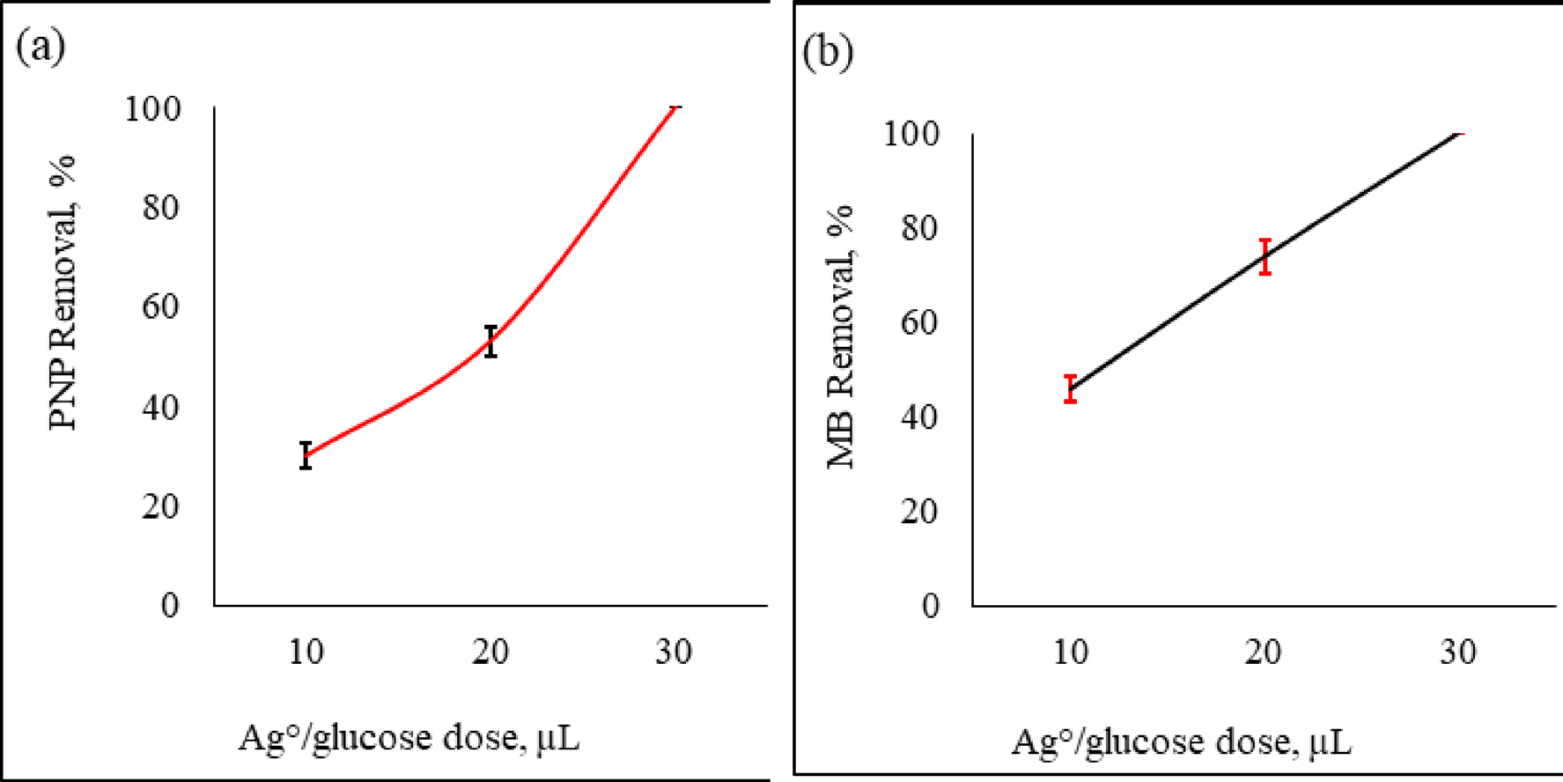
Effects of Ag°/glucose doses on PNP (**a**) and MB (**b**) removal percentage at room temperature. (contact time: 30-minute, Ag°/glucose dose: 10, 20, and 30 as µL for each one milliliter sample, and initial pollutant concentration 10 µg/mL). The error bars represent the standard error of the mean.

The operating conditions were: pollutant concentration is 10 µg/mL, contact time is 30 min, and temperature is maintained at the ambient room temperature (22° C). The Ag°/glucose nanoparticles doses were 10, 20, and 30 µl/mL from the liquid formula. Increasing Ag°/glucose dose from 10 to 30 µl/mL raised the PNP removal percentages from 30 up to 100% (Fig. 2a) and from 46 to 100% when MB is the target (Fig. 2b). The earlier research clarifies increasing in pollutant removal percentage with increasing Ag°/glucose dose occurred because a greater mass provided more active sites for bio-sorption^43,44^. Also, increasing the Ag°/glucose dose may be causing a rise in active surfaces that are available for use as adsorption sites^45,46^. From the data in the current section, 20 µL/mL Ag°/glucose liquid formula was chosen as the adsorbent dose for further experiments.

### Effects of the contact time on the removal potency

**Fig. 3:**
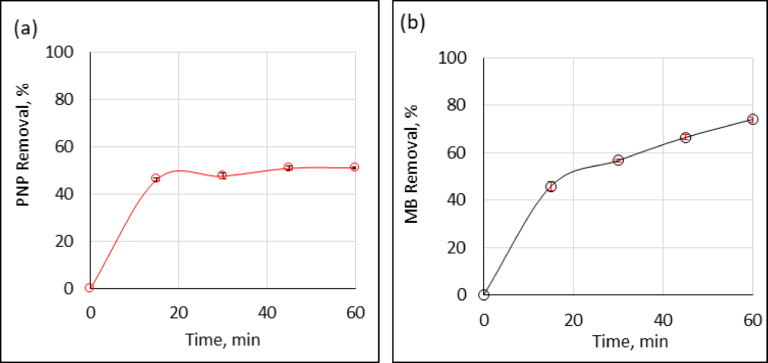
Effects of contact time on the removal percentage of PNP (**a**) and MB (**b**) at room temperature. (contact times:15, 30, 45, and 60 min, Ag°/glucose dose:20 µL/mL, and initial pollutant concentration:10 µg/mL). The error bars represent the standard error of the mean.

The operating conditions were: pollutant concentration is 10 µg/mL, contact time from 15 to 60 min, Ag°/glucose dose is 20 µL/mL, and temperature maintained at the ambient room temperature. During the first 15 min, the optical density of the PNP (Fig. 3a) and MB (Fig. 3b) solutions decreased remarkably because of the addition Ag°/glucose nanoparticles to the target contaminated samples. Thus, the incubation of pollutants for 15 min with Ag°/glucose would be the optimum contacting time and will be used for the remaining experiments. A lot of studies were conducting to assess the catalytic potentials of silver nanoparticles and they concluded that, PNP could be rapidly decreased by green silver nanoparticles^4^ also 98.3% of PNP was eliminated in 11 min when green nano silver was utilized^25^ silver nanoparticles have a useful catalytic ability to degrade and reduct PNP in the presence of aqueous sodium borohydride^35^ and in less than 5 min of incubation with silver nanoparticles greenly synthesized, by *Hordeum vulgare* L. could be suitable to eliminate the same two tested pollutants either in single or mixed states^20^.

### Kinetic studies

**Fig. 4:**
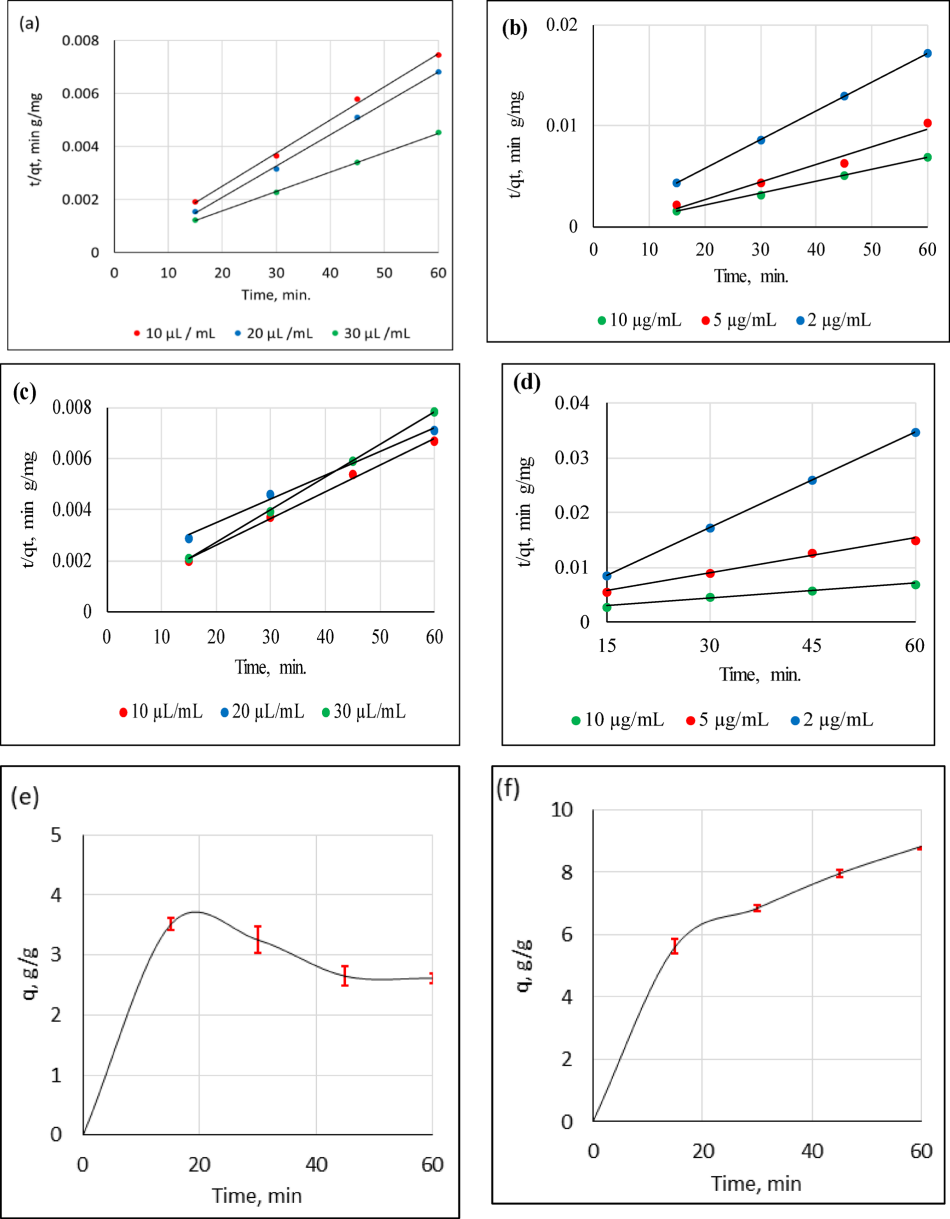
Pseudo-second-order plot: (**a**) effect of Ag°/glucose dose on PNP kinetic parameters, (**b**) effect of PNP concentration on PNP removal kinetic parameters, (**c**) effects of Ag°/glucose dose on MB kinetic parameters, and (**d**) effect of MB concentration on MB removal kinetic parameters. The dots represent the experimental data however the lines represent the Pseudo-second-order adsorption kinetic model. (**e**) Effects of time on the adsorption capacity of PNP (g/g), (**f**) Effects of time on the adsorption capacity of PNP (g/g). The error bars represent the standard error of the mean.

Removal rates of the studied pollutants and their controlling mechanisms were examined at different ratios of sorbed/sorbent as part of the kinetics studies. Pseudo-first-order, pseudo-second-order, Elovich, and intra-particle diffusion kinetic models were employed to describe the mechanism of *p-*nitrophenol and methylene blue removal by Ag°/glucose. The parameters obtained for the pseudo-first-order, pseudo-second-order, Elovich, and intra-particle diffusion kinetic models are presented in Table 2; Fig. 4a- f. The operating conditions used for the two target pollutants were Ag°/glucose doses of 10, 20, and 30 µl/mL; target pollutant (PNP or MB) concentrations of 2, 5, and 10 µg/mL; intervals ranging from 15 to 60 min; and temperature maintained at the ambient temperature.

The effects of Ag°/glucose concentrations (10, 20, and 30 µl/mL) on the PNP and MB kinetic parameters are presented in Fig. 4 (a & c), respectively, and Table 2. Depending on the R^2^ values, the most efficient results were recorded when the Ag°/glucose dose was 20 µl/mL. At such dose (20 µl/mL), pseudo-second order was the best fit kinetic model for the two tested pollutants (PNP and MB). Additionally, the similarities between the experimentally determined and calculated q values confirmed that the pseudo-second-order model effectively described the PNP and MB equilibrium kinetics.

The same kinetic mechanism was confirmed when PNP or MB was applied at different concentrations (2, 5, and 10 µg/mL), Table 2; Fig. 4 (b & d). These findings confirmed that the best-fit kinetic model was a pseudo-second-order model. The reliability of the kinetic models was determined by the coefficient value (R^2^), where the best fit model must have a R^2^ value closest to 1. Furthermore, the model effectively describes equilibrium kinetics, as evidenced by the similarities between the computed and experimental data^1^ as illustrated in Fig. (4 e & f). The sorption of PNP and MB onto Ag°/glucose appears to be limited by chemical sorption, which is influenced by the adsorbent’s active sites at room temperature, as indicated by the rise in total PNP and MB sorption in the pseudo-second-order model^47^. According to this model, which is consistent with the current data, a rapid response eventually finds equilibrium, followed by a sluggish reaction that can go on for a long time^48^. Many studies have been conducted to determine the best kinetic model for describing the absorption of PNP and MB, for example, for the absorption of MB by agar/κ-carrageenan hydrogels^49^ for the removal of PNP using activated biochar^47^and for the removal of PNP-MB mixtures by green silver nanoparticles^20^. These studies confirmed that the pseudo-second order is the most suitable kinetic model for removing MB and PNP, which is in agreement with the current findings. Furthermore, when the value of computed α > β in the Elovich model, the adsorption process proceeded more quickly than the desorption process^50^ which is consistent with our results (Table 2).

**Table 2:** Kinetic parameters of PNP and MB adsorption on Ag°/glucose.

Kinetic Models	Unit	PNP			MB		
Ag°/glucose dose, μL/mL		10	20	30	10	20	30
*q*_*e* experimental_		8231	9687	13,240	8980	8486	7646
Pseudo-First order	*q*_*e* calculated_	19.03	63.85	----	2201.51	6553.80	----
	K_1_	0.031	0.048	----	0.030	0.044	----
	R^2^	0.034	0.762	----	0.992	0.98	----
Pseudo-Second order	*q*_*e* calculated_	10E + 03	10E + 03	14,286	10E + 03	10E + 03	10E + 03
	K_2_	0.001	3E-05	5E-05	2E-05	5E-06	5E-05
	R^2^	0.998	0.999	1	0.998	0.991	1
Elovich	Β	51.77	709.76	810.17	5520	2308	404.7
	α	5E + 63	2E + 04	3E + 02	0.008	0.001	8493
	R^2^	0.02	0.88	0.78	0.776	0.985	0.776
Intra-particle diffusion	K_diff_	14.35	258.45	275.55	353.58	838.5	137.64
	C	7888	10,732	11,324	6157.3	1975	6689
	R^2^	0.013	0.897	0.691	0.976	0.999	0.691

Kinetic Models	Unit	PNP			MB		
Pollutant concentration, μg/mL		2	5	10	2	5	10
*q*_*e* experimental_		3489	7119	9687	1726	3977	8486
Pseudo-First order	*q*_*e* calculated_	–	39	64	–	2E + 03	6554
	K_1_	–	0.0536	0.0478	–	0.0378	0.0435
	R^2^	–	0.848	0.762	–	0.994	0.980
Pseudo-Second order	*q*_*e* calculated_	3E + 03	5E + 03	1E + 04	1.7E + 03	5E + 03	1E + 04
	K_2_	9E + 09	5E-05	3E-05	1.2E + 10	1.5E-05	5E-06
	R^2^	1	0.972	0.999	1	0.993	0.991
Elovich	β	7 E-12	588.7	709.76	3E-12	916.05	2308
	α	0	5E + 03	2E + 04	–	1.3E-03	7E-04
	R^2^	–	0.349	0.879	–	0.985	0.985
Intra-particle diffusion	K_diff_	–	235.19	258.45	9 E-13	330.94	838.5
	C	–	8139.3	10,732	1726.4	1392.4	1975
	R^2^	–	0.428	0.897	–	0.989	0.999

### Effect of initial pollutant concentrations

**Fig. 5:**
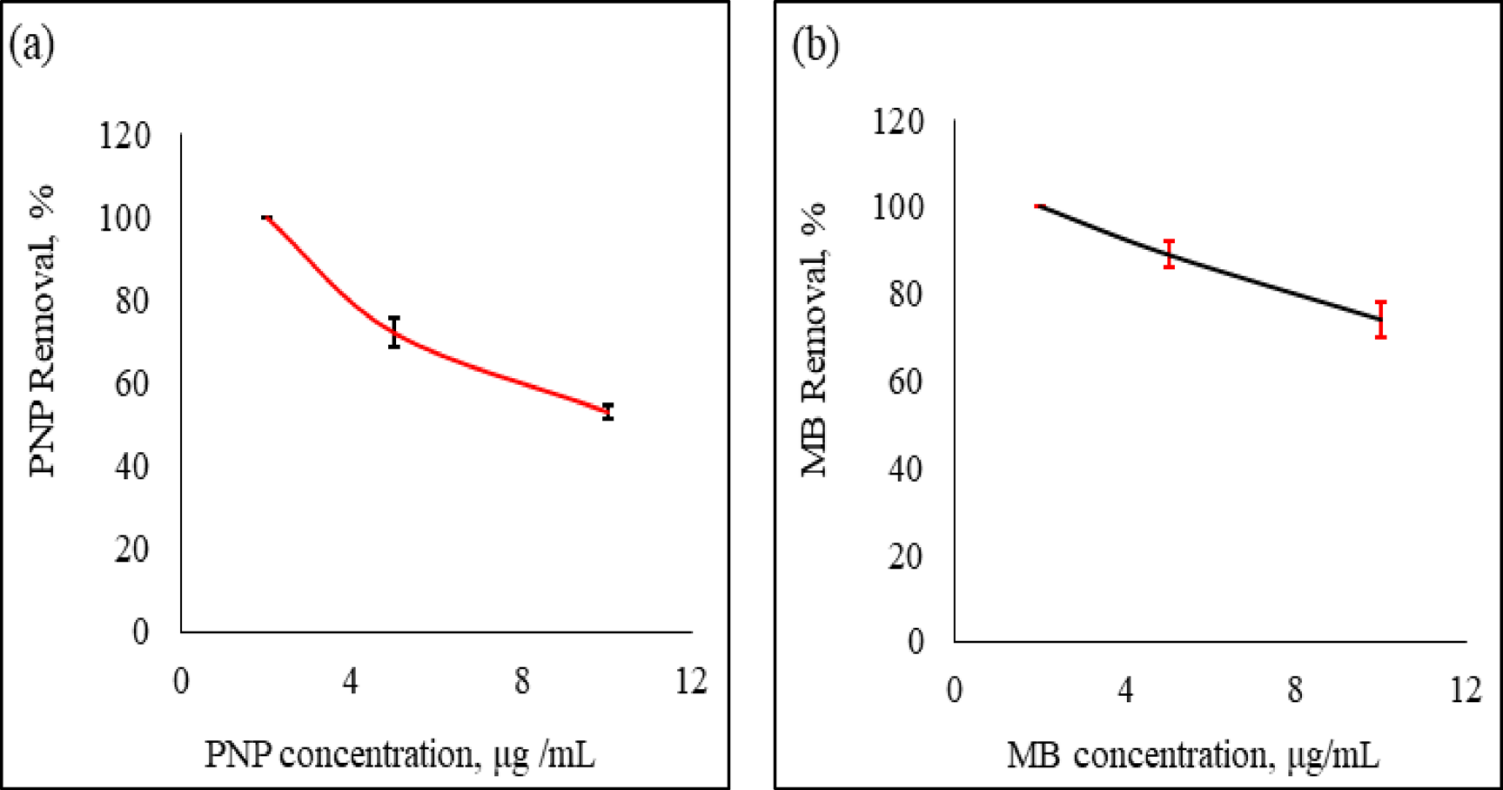
Effects of PNP (**a**), and MB (**b**) initial concentration on the percentage of removal at room temperature. (contact time :15 min, Ag°/glucose dose :20 µL/mL, and initial pollutant concentration :2,5, and 10 µg/mL). The error bars represent the standard error of mean.

The relationship between concentrations of either PNP or MB and their removal percentages are presented in Fig. 5a & b, respectively. The operating conditions were contact time is 15 min, Ag°/glucose dose is 20 µl/mL, pollutant concentrations are 2, 5, and 10 µg/mL, and temperature maintained at ambient temperature. As shown in Figs. 5a & b, when the pollutants concentration increased from 2 to 10 µg/mL, the removal percentages decreased from 100 to 72 and 53% for PNP, and from 100 to 89 and 74% for MB. Similar findings were observed by Hassan et al.,^51^ and they provided the following explanation: at lower initial pollutant concentrations, there were relatively few pollutant molecules compared to the huge number of accessible active sites, which led to a quick uptake by the sorbent. However, a progressive decline in the proportion of removed pollutants was noted when the starting concentrations of pollutants increased; this could be because all possible active sites had been reached.

### Isothermal studies

**Fig. 6:**
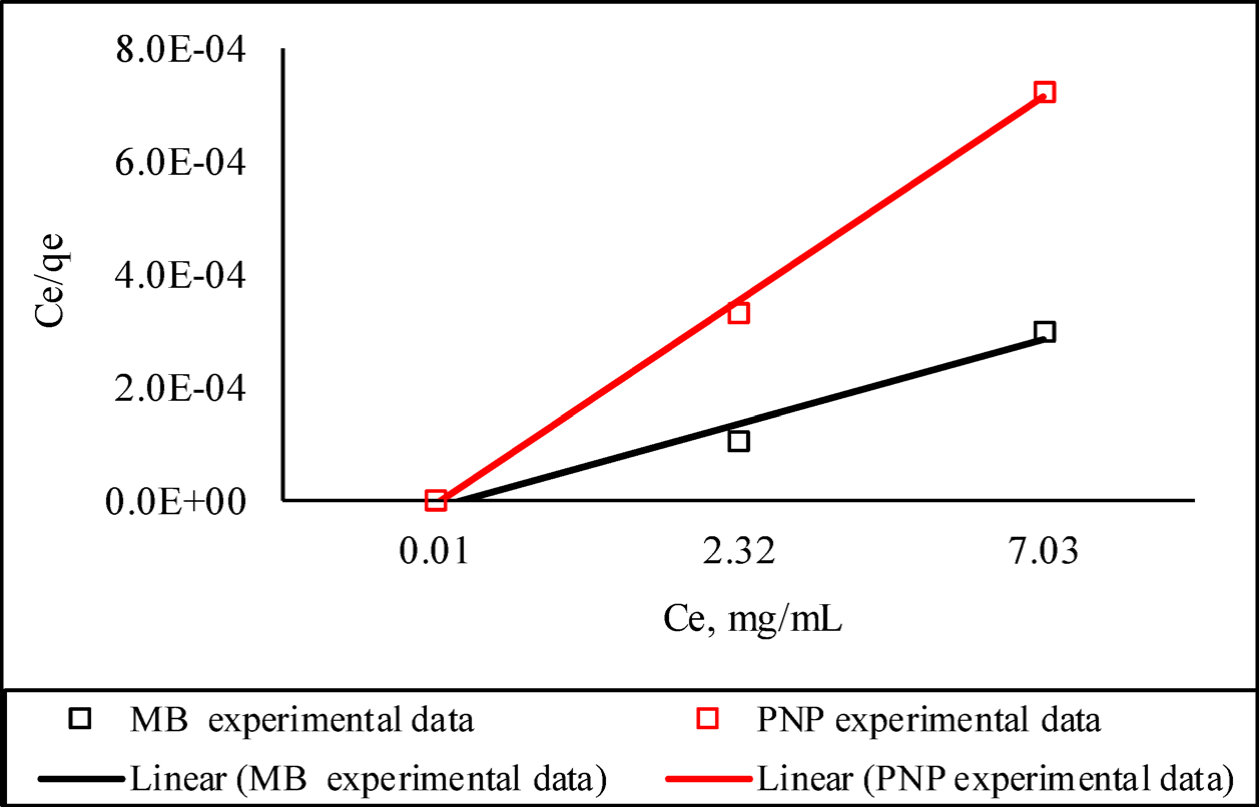
Langmuir adsorption isothermal plot for the adsorption of PNP and MB on Ag°/glucose (Ag°/glucose dose of 20 µl/mL, and initial pollutant concentration of 2 to 10 µg/mL).

An explicit examination of the relationship between the concentration and sorbed amount of PNP or MB was done using the Langmuir, Freundlich, and Tempkin isothermal models. Parameters that were obtained from the plots are displayed in Table 3; Fig. 6. Based on the correlation coefficient (R^2^), the model that best explains the adsorption process was selected. The best-fit model for explaining the adsorption of PNP and MB on Ag°/glucose nanoparticles was the Langmuir isotherm, according to a comparison of R^2^ (1) PNP: Langmuir (0.997) > Tempkin (0.995) > Freundlich (0.958), (2) MB; Freundlich (0.999) > Langmuir (0.971) > Tempkin (0.964). According to an analysis of the Langmuir plot, the maximum adsorption capacity (*q*_max_) for Ag°/glucose being 2500 and 10,000 mg/g for PNP and MB, respectively. The value of R_L_ indicates the favorability of the adsorption and can be classified into three ranges: irreversible (R_L_ = 0), linear (R_L_ = 1), or unfavorable (R_L_ > 1)^6^. Based on the current results (Table 3), the adsorption process was beneficial as indicated by the R_L_(PNP) = 0.141 and R_L_(MB) = 0.365.

**Table 3:** Adsorption isotherm parameters of PNP and MB adsorbed on Ag°/glucose.

Isotherm parameters		PNP	MB
Langmuir	q_max_	2.5E + 3	10E + 3
	K_L_	1	0.5
	R_L_	0.141	0.365
	R^2^	0.997	0.971
Freundlich	K_F_	2223	788
	R^2^	0.958	0.999
Temkin	K_T_	1.18	1.8
	R^2^	0.995	0.964

### A suggested removal mechanism

**Fig. 7:**
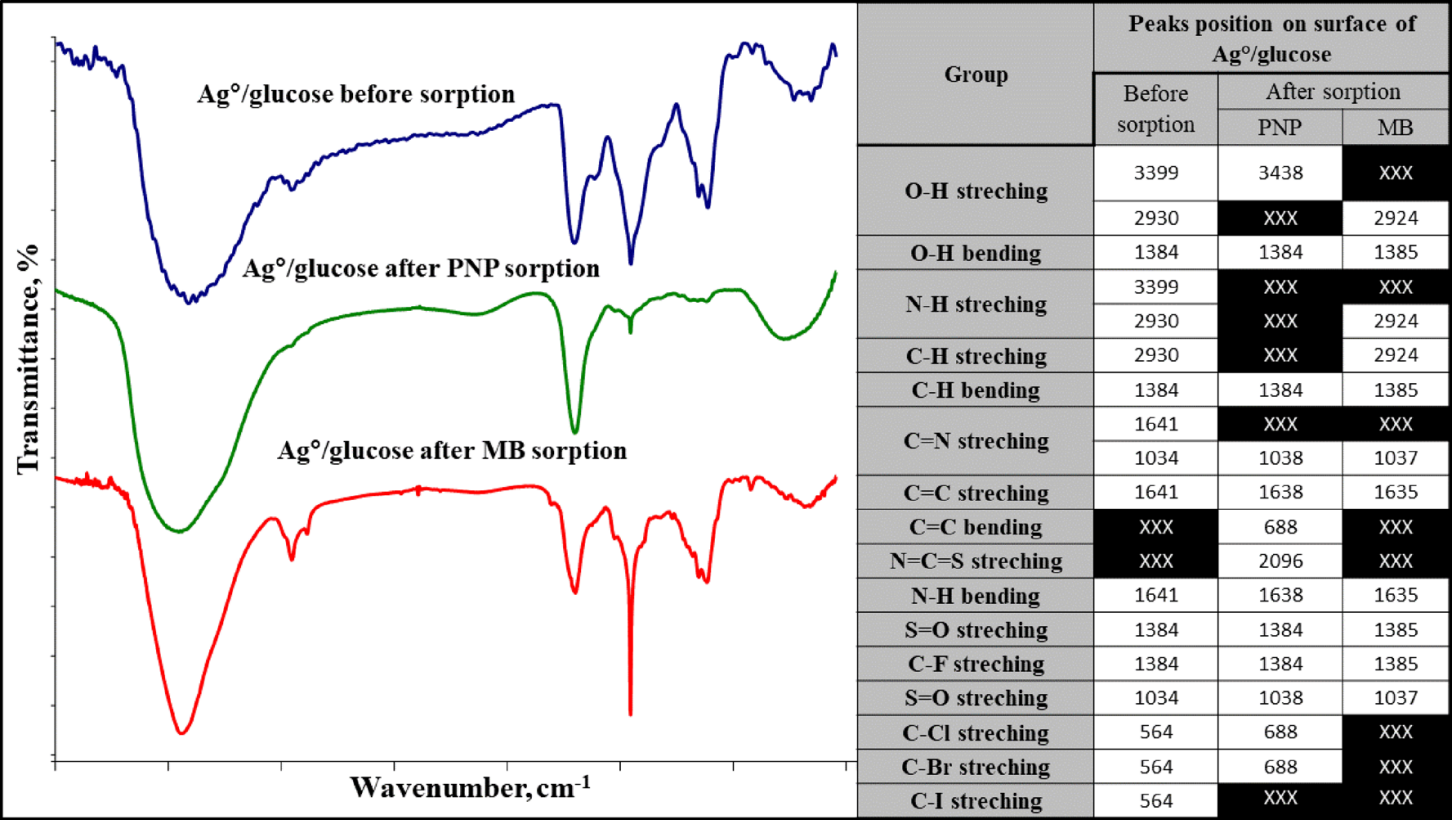
FTIR spectra of the Ag°/glucose formula before and after the remediation process.

**Fig. 8:**
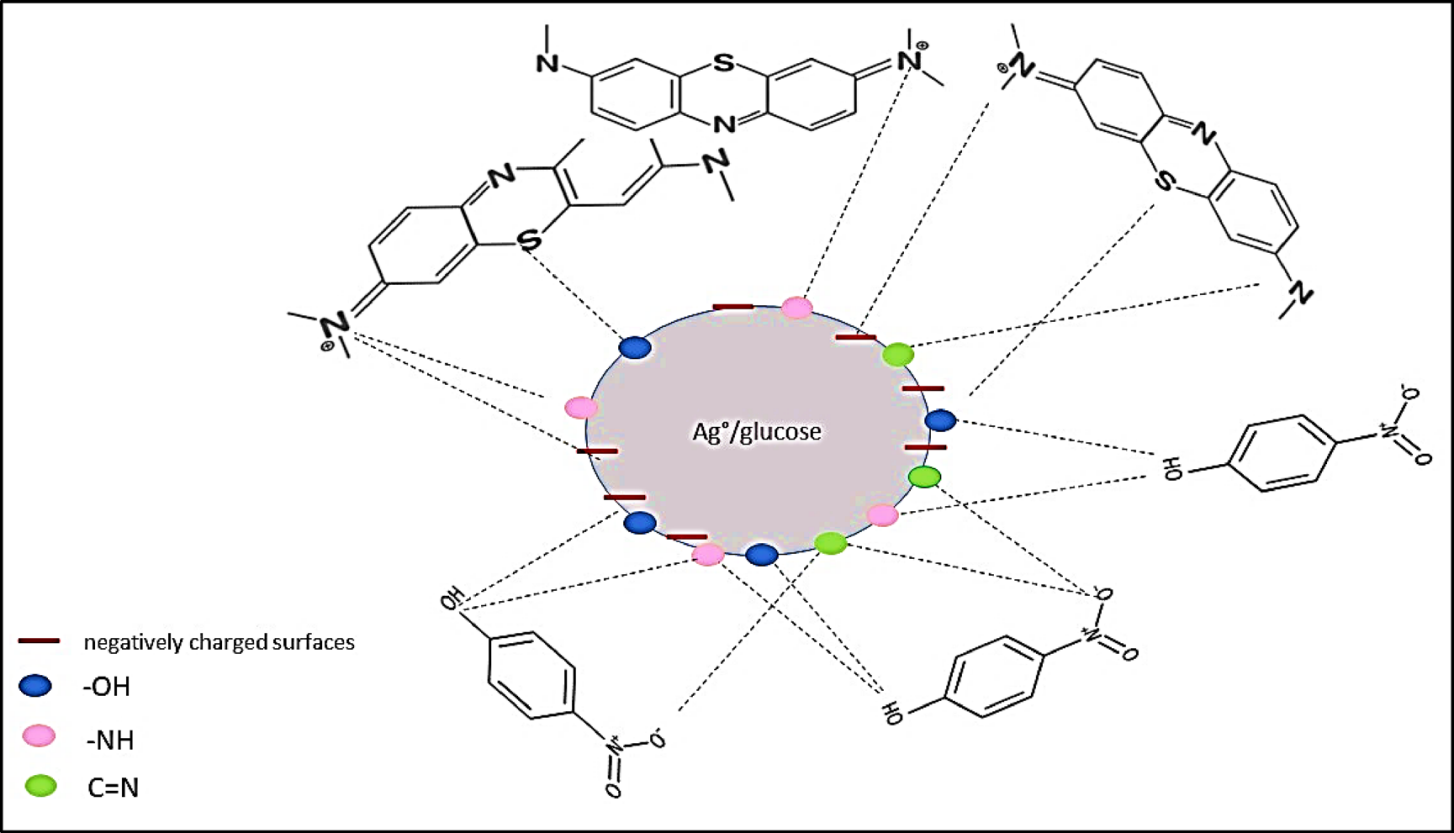
The suggested scheme of PNP and MB adsorption mechanism onto Ag°/glucose nanoparticles.

The impact of the interaction between the Ag°/glucose functional groups and the sorbed pollutants was investigated using FTIR analysis before and after sorption (Figs. 7 and 8). It was found that the Ag°/glucose’s primary functional groups were 3879.83, 3846.88, 3725.18, 3398.68, 2929.67, 1640.66, 1383.90, 1075.44, 1034.11, and 563.68 cm⁻¹. Hence, after PNP sorption, some peaks that represent O-H, N-H, C-H, C= N, and C-I groups at 2930, 3399, 3930, 1640.66, and 563.68 cm-¹ disappeared. This is explained by the structure of PNP, which has both a hydroxyl group and a nitro group on opposite sides of the benzene ring. These groups can form hydrogen bonds with oxygen-containing functional groups (like -OH groups) on the Ag°/glucose surface^52^. As well as the nitrogen atom in the N-H group on the Ag°/glucose surface can act as a hydrogen bond acceptor, while the hydrogen atom in the -OH group of PNP can act as a hydrogen bond donor^53^. Also, at certain pH levels, PNP can exist as a negatively charged phenolate ion. This can lead to electrostatic interactions with positively charged sites on the Ag°/glucose surface, including those associated with C = N groups^54^. Finally, the iodine atom in the C-I bond can be displaced by the oxygen atom of the phenolic group in p-nitrophenol through a nucleophilic aromatic substitution reaction^55^. In the case of MB sorption, peaks representing O-H, N-H, C =N, C=C, N=C=S, C-Cl-, C-Br, and C-I disappear. Because MB is a cationic dye, when dissolved in water, it dissociates into a positively charged ion, and Ag°/glucose with negatively charged surfaces or functional groups containing nitrogen (like amines or amides) can attract and bind to the MB^56^. Additionally, the hydroxyl groups on the Ag°/glucose nanoparticles surface can form hydrogen bonds with MB molecules. This involves the sharing of hydrogen atoms between the hydroxyl group and specific atoms (nitrogen and sulfur) in the MB molecule^57,58^. Likewise, the nitrogen atom in the imine group (C= N) can act as a hydrogen bond acceptor, creating a hydrogen bond with hydrogen atoms in the MB dye^59^. Finally, the interaction of the sorbed component (PNP and MB) with the active sites of the sorbents (Ag°/glucose) might be responsible by hydrogen bonding, electrostatic contacts, the π–π interaction, and the hydrophobic interaction are the primary suggested interactions between PNP, MB, and Ag°/glucose, as seen in the FTIR scheme^60^.

### Lab scale cost

From an economic standpoint, estimating the cost of producing Ag°/glucose is crucial. Consequently, the lab scale cost performance for synthesizing 100 milliliters of the current nanomaterial is summarized in Table 4. Herein, we must consider the following points:

- The Ag°/glucose using in traces doses (20µL/mL).
- The maximum adsorption capacity value was 2.5E + 3, and 10E + 3 mg/g for PNP and MB, respectively.
- The possibility for reuse and regeneration and its positive impact on the coast will be examined in subsequent research.

We can summarize that the cost of remediating five liters containing 10 mg/L from PNP or MB by Ag°/glucose is almost equal to ~ 20 $. In comparison to the cost of poisonings, particularly those that occur in low- or middle-income countries or that affect children, this expense is negligible. According to one South African study, hospitalization for poisoning costs at least US$1.4 million annually in direct expenses^61^. Medical treatment accounted for nearly 9% of the nearly $400 million lifetime cost of poisonings in children under the age of fifteen. Including medical costs, lost earnings, and lost quality of life, this yields a conservative estimate of US$ 1780, on average, for each poisoning case^62^.

**Table 4:** Lab scale cost estimation.

Material	Amount (g)	Approximate Cost ($.)
AgNO_3_	5.60571	20
Gelatin	0.396	0.02
Glucose	13.2	0.1
NaOH	1.32	0.02
Electric energy	Almost 80 °C for one hour	0.02
Total expected costs for synthesis a 100 ml of Ag°/glucose		20.16

###  Comparative analysis with other greenly synthesized nanoparticles

Table 5. summarized a comparison between Ag°/glucose and other greenly silver nanoparticles according to their sources, characteristics, and catalytic efficiencies.

**Table 5:** A comparison between ag°/glucose and other green silver nanoparticles.

Adsorbent	Plant extract / Base	Size (nm)	Zeta potential (mv)	Target	q_max_ (mg/g)	Kinetic model	Refs
*Om-*AgNPs	*Ophiorrhiza mungos*	17	–	MB	80.451	PSO	^57^
CuO NPs	*Melia azedarach* fruit	20–40	-30.4	MB	26.738	PSO	^63^
UL-AgNPs	*Urena lobata* leaf extract	20	–	MB	218.95	PFO	^64^
*Malva*-AgNPs	*Malva parviflora* leaf	100 ± 1	− 26.4	MB	400	PSO	^65^
PVA/INPs	green tea	22	− 30.4	MB	24.509	PSO	^66^
**The current formula**	**Glucose**	**21 to 31**	**− 16**	**MB**	**10E + 3**	**PSO**	**Present work**
				**PNP**	**2.5E + 3**		
AgNPs/AC	*Eragrostis plana* Nees	8.5 ± 1.4		PNP	140.19	PSO	^67^
CaAl-LDH/g-CN@Fe3O4	Graphite carbon nitride	150–200	–	PNP	2500	PSO	^68^
CHAC-250	Corn husk	200–300	–	PNP	9.930	PSO	^69^
CHAC-500	Corn husk	100–200	–	PNP	11.5	PSO	^69^
10% Fe-MCA	Resorcinol	10.93	–	PNP	141	PSO	^70^

### Novelty and future scope of the current study

The introduced *q*_max_ of the studied pollutants is extremely high and enables the current formula Ag°/glucose to be used on a large-scale wastewater remediation, even for different categories of pollutants. In the current study, the catalysis Ag°/glucose was used as an aqueous colloidal solution in a laboratory experiment to get a primary understanding of its efficiency as a catalyst. The priority in further studies is to immobilize the Ag°/glucose on an eco-friendly carrier and transfer it to a manufacturing scale.

## Conclusions

This study introduces a promising green silver nanoparticle (Ag°/glucose) as a tool to remediate PNP or MB from synthetically contaminated water. From the presented data, it can be concluded that the features of Ag°/glucose are as follows: (1) simple and fast synthesis method. (2) distinctive characterizations (λ _max_ at 430 nm, 21 to 31 nm diameter, and zeta potential equal to -16 mV). (3) time required for removing PNP and MB was observed as fifteen minutes when 20 µL/mL of Ag°/glucose was applied. (4) the pseudo-second order model describes adequately the kinetic data. (5) the Elovich model suggests that the initial rate constant was greater than the desorption constant for both PNP and MB. (6) Langmuir and Freundlich isotherm models provides the good correlation for the PNP and MB equilibrium data, respectively. (7) the R_L_ value was lower than one for both target pollutants. According to the research, Ag°/glucose has the potential to be an advantageous resource for treating wastewater, particularly when it comes to the organic materials pollutants.

## Data Availability

Availability of data and materials: The authors confirm that the data supporting the findings of this study are introduced and available within the manuscript.
